# Perceptions of diabetes distress during pregnancy in women with type 1 and type 2 diabetes: a qualitative interpretive description study

**DOI:** 10.1186/s12884-024-06370-w

**Published:** 2024-04-03

**Authors:** Holly Tschirhart, Janet Landeen, Jennifer Yost, Kara A. Nerenberg, Diana Sherifali

**Affiliations:** 1 School of Nursing, Faculty of Health Sciences, McMaster University, Hamilton, Canada; 2 M. Louise Fitzpatrick College of Nursing, Villanova University, Pennsylvania, USA; 3 Departments of Medicine, Obstetrics & Gynecology, and Community Health Sciences, University of Calgary, Calgary, Canada

**Keywords:** Diabetes mellitus, type 1, Diabetes mellitus, type 2, Pregnancy, Interpretive description, Qualitative research, Diabetes distress

## Abstract

**Background:**

Diabetes distress is commonly seen in adults with pre-existing diabetes and is associated with worsened glycemic management and self-management practices. While a majority of women report increased stress during pregnancy, it is unknown how women with type 1 or type 2 diabetes experience diabetes distress during this unique and transitional time.

**Purpose:**

This study aimed to understand the experiences and perceptions of diabetes distress in women with pre-existing diabetes during pregnancy.

**Methods:**

A qualitative study using an interpretive description approach was conducted. In-depth, one to one interviewing was used to capture rich descriptions of the pregnancy experience. Nested, stratified, and theoretical sampling was used to recruit 18 participants with type 1 and type 2 diabetes from the quantitative strand of this mixed methods study. Constant comparative analysis was used to inductively analyze the data and develop themes.

**Findings:**

Four themes, each with several subthemes, emerged under the main finding of “Diabetes Distress”: 1) Worry for Baby’s Health – “What’s this going to do to the baby?”’ 2) Feeling Overwhelmed with Diabetes Management—“It just seemed unattainable”; 3) Living with Diabetes – “There’s no way out” and 4) Cycle of Diabetes Distress.

**Conclusions:**

The findings from this study identify the sources and experiences of diabetes distress during pregnancy in women with pre-existing diabetes. Diabetes distress often presents as cyclical and multifaceted during pregnancy, with elements of fear for the unborn baby, difficulties with diabetes management, and having negative lived experiences of diabetes. Further work is needed to develop appropriate screening tools for pregnancy and interventions to mitigate diabetes distress. Diabetes educators are well-positioned provide emotional support and person-centred self-management education to individuals with diabetes.

## Background

For women with pre-existing diabetes who become pregnant, diabetes management will be at the center of their pregnancy journey. Pre-existing diabetes is type 1 diabetes (T1D) or type 2 diabetes (T2D) that is present before pregnancy and pregnancy in these populations is considered high-risk for mother and baby [1, 2]. Women with pre-existing diabetes may enter pregnancy with pre-existing complications of diabetes such as retinopathy, chronic kidney disease, and peripheral neuropathy that require management [1]. Managing glycemia in women with pre-existing diabetes during pregnancy remains a critical element of care to reduce the risks to the fetus caused by elevated blood glucose [3]. These risks require women to prioritize blood glucose management throughout pregnancy to avoid neonatal complications such as congenital anomalies, miscarriage, stillbirth, preterm birth, and macrosomia [1, 4].

While adoption of long acting insulin analogues and continuous glucose monitoring has improved glycemic management [5, 6], optimizing glycemic targets remains a challenge for many women. Current Canadian guidelines recommend that women with pre-existing diabetes conduct self-monitoring blood glucose (SMBG) a minimum of seven times per day, and glucose targets are set to < 5.3 mmol/L at fasting and before meals, < 7.8 mmol/L at one hour postprandial, and < 6.7 at two hours postprandial [1]

Critically, the incidence of pre-existing diabetes in pregnancy has risen over the past two decades, as a result of increasing numbers of women with T2D, due to advancing maternal age and obesity [7–9]. In one Canadian study, the rate of pre-existing diabetes in pregnancy doubled between 1996 and 2010 (0.7–1.5%, *P* < 0.001) [10], trends that were mirrored in the United States [8, 11] and United Kingdom in that same period [9]. More recent estimates suggest that in high-income countries, pre-existing diabetes occurs in seven to 15 per 1000 pregnancies [12, 13]. A population-based cohort study in Ontario, Canada found the rate of pre-existing diabetes in pregnancy significantly increased in non-Indigenous women from 13.9 to 20.5 per 1000 deliveries from 2002 to 2015 (*p* < 0.001), while the rates for Indigenous women were 40.5 per 1000 deliveries in 2015 [14]. Considerations for this increasing and changing demographic include less frequent exposure to preconception planning and a shorter duration of the condition in women with T2D [15], which may lead to less preparedness for the intense self-management requirements of pregnancy [16].

Previous literature sought to understand the patient experience during this specific time of pregnancy. Qualitative studies have described frequent psychosocial and emotional stresses during pregnancy with pre-existing diabetes. The high level of diabetes management has been reported to make women with pre-existing diabetes feel overwhelmed and impacted their ability to enjoy their pregnancy [17]. Findings by Singh et al. [18] suggest that coping with a high-risk pregnancy contributes to significant psychological distress, including feelings of vulnerability, anxiety, worry, uncertainty, and guilt. Two qualitative studies described themes suggesting emotional and psychosocial issues during pregnancy. A phenomenological study of 14 Swedish women with T1D identified the themes of loss of control, perception of having an “unwell*”* body, and frequent blame, pressure, and worry for the health of their baby [19]. Interviews from another qualitative study of pregnant women with T1D at a high-risk antenatal care unit revealed that due to concerns about bringing the baby to term, women felt hopeless and distanced themselves from their pregnancy [20]. The women interviewed also reported feeling sorrow and loss because they did not feel like average pregnant women [20].

Diabetes distress (DD) is a well-established phenomenon for adults with diabetes, describing the emotional burnout that individuals living with this chronic condition experience [21, 22]. One multinational study of adults with T1D and T2D found the prevalence of DD as high as 44% in a sample of nearly 8500 individuals across 17 countries. In Canada, the DD prevalence (representing three provinces) was 28% [23]. Importantly, women may be more likely to experience DD, as females with T2D were three times more likely to report DD over 18 months in a community-based longitudinal study [24]. In non-pregnant adults with pre-existing diabetes, DD is closely related to glycemic management and diabetes management [25]. It is also significantly associated with worsened blood glucose manangement and diabetes self-management [25, 26]. Individuals with high DD levels are at a significantly increased risk for low self-care [27], decreased quality of life [28], and lower adherence to treatment [29].

In interviews with young adults with T1D recruited from Irish Facebook support groups, themes of DD related to pregnancy conception, progression, and delivery was present before conception in women with T1D [30]. Seeking to understand the sources of DD in young adults with T1D, concerns about pregnancy were a common source of DD in 12 of the 29 female interviewees who were between ages 23–30. The participants cited worries about miscarrying; conception taking place during a time of unstable glycemic management; and the health implications for both themselves and the baby if the infant was large for gestational age [30]. While the objectives of the study were specific to DD, the female participants commenting on pregnancy as a cause of distress were not experiencing pregnancy themselves at the time they were interviewed [30].

A meta-synthesis of qualitative research on the experience of distress, depression, and anxiety during pregnancy in the general pregnancy population described a subtheme of psychosocial factors called an emotional response, which described the expression of fear and anxiety for unknown fetal and pregnancy outcomes; the existing stress from living with a chronic condition exacerbated by the pregnancy; and feeling that pregnancy with a chronic condition is emotionally complex [31]. While the qualitative literature on diabetes and pregnancy has uncovered themes that mirror elements found in the construct of DD, including descriptions of anxiety and stress related to their diabetes management, it is unknown if and how women with pre-existing diabetes experience DD and if and how it affects their pregnancy experience and quality of life. Women with pre-exisiting diabetes during pregnancy face rigorous self-management requirements and risks during pregnancy, which may contribute to increased stress and other negative emotions, such as DD. Therefore, healthcare providers need to have a better understanding of the individual’s perspectives on the impact that DD has on their experiences of pregnancy and diabetes self-management as it is important to optimize pregnancy outcomes.

The aim of this study was to extract detailed descriptions about the experiences of living with pre-existing diabetes in pregnancy in order to understand how women perceive DD during such an important time in their lives. Using qualitative methodology, the researchers sought to answer the study question: *How do women with pre-existing diabetes receiving obstetrical care at a high-risk clinic in Southern Ontario perceive diabetes distress during pregnancy?*

A companion paper will address the second study question [32]: *How does the experience of varying levels of diabetes distress impact pre-existing diabetes management in pregnancy?*

## Methods

### Design

This study was the second strand of a sequential explanatory mixed methods study and used an interpretive description approach. Interpretive description was chosen because it is a method that creates knowledge relevant to the clinical context of applied health disciplines, making the findings relevant to clinical practice [33].

### Participants and sampling

All participants provided informed consent before taking part in any study-related activities. Nested sampling was first conducted to re-recruit the same participants who had taken part in the initial quantitative strand (cross-sectional study) and who had previously provided written consent to be contacted for the second strand (qualitative study) [34]. The study population was recruited from a high-risk pregnancy clinic at a tertiary care center that ran a once-weekly diabetes and pregnancy clinic for women with T1D and T2D. For the initial and second strand, the inclusion criteria were females with diagnosed T1D or T2D over the age of 18 years; currently or had previously been a patient of the local high-risk pre-existing diabetes in pregnancy clinic; if they were currently pregnant they could be any gestational age and there was no limitation to how far in the postpartum period they could be.

For the second (qualitative) strand, stratified sampling was then applied to capture variations in DD and allow comparison across subgroups. Stratified samplng was achieved by stratifying the cohort of participants from the quantitative strand by DD scores (high/positive and low/negative) and diabetes type. Scores for DD were measured using the Problem Area in Diabetes (PAID) scale. The first interviews were purposely initiated with women from both the categories of high score/positive for DD (defined by a PAID score ≥ 40) and low score/negative for DD (defined by a PAID score ≤ 40) and included both T1D and T2D. Following the analysis of these initial interviews, theoretical sampling took place intending to elaborate on emerging concepts/constructs as they were uncovered during the interviews.

The purpose of stratified sampling is to showcase subgroups and allow comparison across subgroups [35, 36]. Stratified sampling was congruent with the qualitative study question because it captured variations in the phenomenon of interest of DD with categories of high score/positive for DD and low score/negative for DD. After stratified sampling with the initial interviews, theoretical sampling was used to sample women based on constructs that emerged from the data. Data saturation was met once the research team determined that the data signalled comprehensive and adequate information to answer the qualitative questions.

### Data collection

In-depth, one-to-one qualitative interviews were conducted with participants by the same researcher (HT). A semi-structured interview guide that had been informed by cross-sectional study results from the first strand of the mixed methods study was used and adapted throughout interviewing and initial data analysis. Participants were asked about their experiences of stress and DD in pregnancy, challenges and successes managing their pre-existing diabetes during pregnancy, and experiences with clinical care and social support during their pregnancy. Seventeen interviews were completed over the telephone and one interview was conducted in person at the request of the participant on-site after her obstetrical visit. All interviews lasted approximately one hour and were audio recorded. Interviews were then transcribed verbatim with any personal information de-identified.

### Data analysis

Data collection and analysis were concurrent and iterative. Two members of the research team (HT and DS) reviewed all the transcripts and made analytical memos to capture themes, emerging patterns, questions, and other pertinent elements about the data and phenomenon under study to refine the ideas that led to analytical conclusions [33]. Consensus of all researchers was the achieved on how the data was initially categorized and if there were emerging concepts that warranted new questions being added to the interview guide.

Constant comparative analysis, a type of inductive analysis, was conducted using NVivo 11 software and involved reviewing the transcribed interviews, creating codes, grouping codes into categories, and finally creating themes from the categories [37]. As codes and themes developed, researchers DS and JL reviewed transcripts and confirmed the findings. Throughout the process, new data was compared to emerging themes, followed by examining individual cases, similar and contrasting cases, and the broad dataset to uncover and test patterns and relationships.

### Rigor

Several strategies were employed throughout the study to ensure the trustworthiness and credibility of data analysis. The thoughtful clinician test was used to share emerging themes and interpretations created by the research team clinicians working in the high-risk pregnancy clinic to obtain feedback if the findings were consistent with their experiences providing care for women with pre-existing diabetes in pregnancy. There was a prolonged time in the field completing eighteen interviews of approximately one hour long. Triangulation was achieved by verifying the data sources of interview notes against interview transcripts [35] and triangulating researcher and stakeholder interpretations and experience against the data by using the thoughtful clinician test described above [37]. The concurrent data collection and iterative analysis also served as a verification method for trustworthiness in this study. An audit trail was kept of the study processes and details to allow auditing and replication and attest to researcher objectivity [35]. To help to track the evolution of the data over time, thorough documentation in the form of field notes, reflexive journal entries, interview guides and recorded interviews, coding workbook, and data analysis notes were kept [33].

### Ethics

All study methods were carried out according to local guidelines and regulations. The Hamilton Health Sciences research ethics board approved this research study (REB #14–222). All subjects provided verbal consent to participate. Digital audio recordings were only accessed by the research team and kept on a password-protected laptop.

## Findings

Eighteen women took part in the qualitative interviews. There were eight women with T1D and 10 with T2D. Participants were between the ages of 19 and 45 and had a mean duration of diabetes of 12 years. Four women were primigravida and 14 were multigravida. All but two women were married or in common-law partnerships. Half of the women were working full or part-time hours. All women had received obstetrical care from a specialized high-risk clinic in Southern Ontario. Participant characteristics are summarized in Table 1.

**Table 1:** Description of participants

Pseudonym	Age	Type of Diabetes	Duration of Diabetes	Gravida/Children	Marital Status	Living Situation
Kelsey	35	T2D	5	3/1	Married	Lives with spouse, children and extended family
Barbara	35	T1D	35	3/2	Married	Lives with spouse and children
Lisa	30	T1D	24.5	2/2	Common-law	Lives with partner and children
Marie	31	T2D	3.5	2/1	Married	Lives with spouse and children
Monique	41	T2D	6.5	3/1	Married	Lives with spouse and children
Saara	30	T1D	22	1/1	Common-law	Lives with spouse and children
Adelaide	31	T2D	6	2/2	Married	Lives with spouse and children
Giselle	32	T1D	23	1/1	Married	Lives with spouse and children
Morgan	30	T1D	19	2/2	Married	Lives with spouse and children
Alex	33	T2D	2.75	3/1	Married	Lives with spouse, children and extended family
Delilah	30	T1D	25	2/1	Married	Lives with spouse and children
Rachel	39	T1D	30	2/2	Common-law	Lives with partner and children
Sammy	38	T2D	3	5/3	Married	Lives with spouse and children
Justine	19	T2D	2	1/0	Single	Lives with parents
Katrina	32	T2D	2.5	3/0	Married	Lives with spouse
Caitlyn	28	T2D	2	1/0	Married	Lives with spouse and extended family
Eileen	45	T1D	6	5/4	Single	Lives with children
Ruth	31	T2D	4	5/3	Married	Lives with spouse, parents and children

### Prelude of thematic findings: time-limited pregnancy journey

To better understand the thematic findings, a pre-understanding of diabetes and pregnancy experience will be summarized here. In interpretive description, researchers are encouraged to use their professional pre-understanding to inform the analysis [33]. Pregnancy is a time of both emotional and physical transition for women with diabetes. While temporary, the period of pregnancy is critical because of the effect glycemic management on fetal growth and development and obstetrical complications at birth [3]. This time-limited journey is defined by diabetes taking on a new form in which it is layered overtop of the pregnancy, creating a multifaceted experience but one that can easily become dominated by the burden of diabetes.

Transition occurs due to three factors: physiological changes to the body, increasing demands to self-manage diabetes, and the medicalization of the pregnancy. From a physical and physiological perspective, women encounter diabetes in a different way than what they were used to before becoming pregnant and could be true for both primigravida women and women who are multigravida with successful pregnancies. There are hormonal changes that affect the action of insulin, greater physical demands to test blood glucose and administer insulin, and challenges determining appropriate insulin doses to reach blood glucose targets [1, 2]. Hypoglycemia may be present to a much greater degree than usual and insulin resistance may lead to atypically large insulin doses. There are also a variety of diabetes self-management responsibilities made up of tasks and skills that women are instructed to master throughout the entirety of pregnancy: reaching glycemic targets, frequent SMBG, adjusting insulin, attending to meal planning and carbohydrate counting, and monitoring by the diabetes team [1, 2].

Depending on the level of engagement and previous glycemic management, pregnancy may be the time a woman devotes the most effort to carbohydrate counting, SMBG, and interacting with diabetes devices such as insulin pumps and continuous glucose monitors. Blood glucose targets, particularly the post-meal targets, are considerably lower than pre-pregnancy glycemic targets. As high-risk patients attending a high-risk obstetrical clinic, women who participated in this study were seen for in-person visits at a higher frequency over the course of pregnancy, increasing from every two weeks at 28 weeks gestation and weekly at 34 weeks gestation. A typical visit would include connecting with multiple providers and repeated ultrasounds, blood work, and non-stress tests may be required throughout. There are also trimester-specific changes with which women will have to cope, including a greater risk of hypoglycemia in the first trimester (particularly for women with T1D), insulin resistance in the second and third trimesters leading to increased insulin needs, and induction planning at the end of the third trimester [1, 2]. Such medicalization of pregnancy, with an emphasis on diabetes tasks and frequent medical visits, combined with physiologic changes further imparts a physical and emotional burden for women.

### Thematic findings

Two main findings emerged from the thematic analysis that were both supported by several themes. The first finding, *Diabetes Distress*, is the subject of this paper and describes how women perceived DD while they were pregnant. Supported by four themes, DD was multifaceted, seen in changing elements of their day-to-day management and closely tied to their unborn baby. The second finding is explored in a companion paper [32].

### Theme 1: Worry for baby’s health –* “What’s this going to do to the baby?”*

Because a healthy baby is the common end goal of each woman’s pregnancy, the worries and concerns for the baby’s health tended to be at the forefront of their minds. Women with diabetes were well-informed about the possibility of negative outcomes, receiving explicit information at the beginning of their care about the various risks to the baby because diabetes is present in the pregnancy. Information about potential risks also came from other sources, including the internet and personal experiences.

#### Fear of infant mortality

In many cases, the participants mentally prioritized what their maternal–fetal medicine team was communicating to them about risks, keeping the most serious outcomes at top of their mind. Ruth described how after receiving information on risks from the care team, the possibility of stillbirth stuck with her:

“Because they tell you all the risks of things that could happen, things that could go wrong. So, you always kind of had that in the back of your head that they say that you could be more at risk for stillbirth. I always just kind of always worried that something could go wrong.” (Ruth, T2D)

For Ruth, the risk of infant mortality was a critical piece of information that translated into persistent worry throughout her pregnancy. Ruth had experienced two miscarriages during her five pregnancies, so her fear was based on her personal experiences with loss.

Similarly, miscarriage was another threat to pregnancy in women with diabetes. Alex (T2D) shared *“Well, now if you Google, and you're not supposed to kind of thing [laughs], sort of extreme things that were nerve racking like the baby could be born blind or I could miscarry. And I had just experienced a miscarriage. And just that the baby itself wasn't going to be healthy or it wasn't going to end up being a viable pregnancy”*. This reflection demonstrated both the alternative sources of information and their power to influence the level of worry someone is experiencing. Alex appeared to understand the consequence of searching the internet for information but still found herself absorbing the negative information that Google provided her. Additionally, Alex illustrated the fear that persists from a previous experience. Having had one recent miscarriage led Alex to fear the past would repeat itself.

The power exerted by a past pregnancy experience could be substantial, demonstrated by a comment from Katrina that showcased the fear that persists following two first-trimester miscarriages which became a time when Katrina was most scared to repeat another loss.

“Going through a miscarriage again I think would just crush me…because the first two are really difficult to get through. So this one, to go through it a third time, I don't know if I would be able to handle it. I was just worried about going through that whole trauma again.” (Katrina, T2D)

#### Fear of the unknown

While the above passages illustrate the specific concerns of infant mortality that women with diabetes had to face, the worry was not always so explicit. Some participants had ambiguous fears about their baby plaguing them. Adelaide reflected on a traumatic first pregnancy experience, which led her to be distrustful about care delivery in her second pregnancy:

“It was just not knowing at that point. In hindsight, I was like, 'Man, you could’ve relaxed, you're good'. I'm just comparing between the two experiences, with so much information the unknown was what worried me.” (Adelaide, T2D)

Adelaide felt a worry about the unknown despite advocating for her care with her second pregnancy and positioning herself in a way that optimizes communication with the team to avoid another traumatic delivery experience. This highlighted the pervasiveness of past trauma which strips women of the control they need to feel secure.

#### Worry about the effect of blood glucose on baby

Measuring and optimally managing blood glucose was prioritized throughout pregnancy. When women observed blood glucose fall out of the prescribed ranges, a new form of worry for the baby’s health took shape as they considered the acute damage of hypoglycemia or hyperglycemia. Caitlyn grappled with concerns over both:

“There’s a lot of little aspects of the pregnancy that make me very anxious. If I get a low in the night, I’m like, how long was I that low? Is this harmful? Or, on the other hand, if I get a ridiculous high and I don't understand why I’m high, but what’s this going to the do the baby as well? There is a definite fear that I don’t think will go away until the baby’s born.” (Caitlyn, T2D)

This passage hints at the frustration Caitlyn felt when at not managing her blood glucose and her uncertainty about the degree of effect an acutely high or low glucose would have had. Even though she was striving to reach blood glucose targets, she could not control every aspect of her blood glucose and their unpredictability. The emphasis on glycemic monitoring and targets made women worried that each reading that was higher or lower than it should be would be harmful to the baby.

One of the consequences of unmanaged blood glucose in women with diabetes is a baby that is large for its gestational age or has macrosomia [1]. Medicalization of the pregnancy takes place as weight is closely monitored. Lisa shared how she discovered the care team had concerns about the baby’s size:

“I think when I first found out, my main concern was baby’s size because she was measuring big from early on. So I think when they first told me 'Okay, she's going to be big, we need to monitor, that's when I started worrying. Why do we need to monitor and what could go wrong from her being so big?” (Lisa, T1D)

Similar to Caitlyn, a knowledge gap about the consequences of a large fetus was exposed and Lisa became stressed by the information about the size of her baby.

### Theme 2: Feeling overwhelmed with diabetes management – *“It just seemed unattainable”*

Women frequently noted feeling overwhelmed with the diabetes self-management requirements of pregnancy, sharing their healthcare providers had high expectations and strict  glycemic targets that could be difficult to achieve consistently. Diabetes management could feel like a burden, with some participants finding it a daily learning curve. In some cases, women reported having little knowledge about the intricacies of pre-existing diabetes in pregnancy, indicating a lack of preconception counseling, which led to them to feel like they did not know what to expect with the changes in self-management.

#### Increased self-care requirements

As self-management of diabetes requires behaviours of self-care, there was suddenly more to do and more to focus on during pregnancy. The physical discomfort of doing more frequent finger pricks throughout the day could make SMBG difficult to perform, as Kelsey explained:

“I think the pricking or testing it four to eight times a day that really got me. Kind of depressed with pricking my fingers. That’s a challenge.” (Kelsey, T2D)

There was also the mental stress associated with testing, which provided feedback to the tester that may be positive or negative. Fueled by a desire to have blood glucose readings in target, a habit of frequent testing and analyzing the numbers can become consuming. Monique describes a fixation with checking her numbers, which led to worry whether the readings were too high when she began using them as a proxy for how the baby was doing at that moment:

“I think it was all mixed in because…the constant monitoring to ensure low numbers fed this this constant worry. And I would analyze those results of my numbers, to give me any indication of the well-being of the baby.” (Monique, T2D)

The dietary aspect of diabetes was also seen as a source of DD. The typical cravings of pregnancy could not be indulged, because of the concern it would shoot up blood glucose. Katrina indicated a sense of restriction in her food choices to maintain glycemic management:

“It has been difficult with some the cravings. And make sure that my blood sugars aren’t crazy. So that's the one thing I found kind of challenging, is having cravings for things and they’re never anything good, of course, they’re always bad things.” (Katrina, T2D)

By labelling foods “*good*” and “*bad*”, this highlighted the strained relationship many women with diabetes have toward food and what carbohydrate-containing foods are acceptable to eat.

#### Mental burden

Typically, women will spend more time on diabetes self-management during pregnancy. The changes to routine and learning curve could be challenging to adapt to. The participants found this extra work was coupled with high expectations and a greater mental burden. This contrasted for Alex against a previous non-diabetic pregnancy and was defined as more frequent appointments and monitoring blood glucose.

“It was definitely more ‘work’ than my first pregnancy when I wasn't diabetic with all of the extra appointments and checking my sugar.” (Alex, T2D)

Alex articulated the heavier workload for someone with diabetes, which included an individual effort as well as more time spent participating in and receiving healthcare.

Katrina had strong internal motivation to achieve good glycemic management and admitted to having high self-expectations, being critical of her food choices when she sees elevated blood glucose:

“When I see the number’s high, I feel like I shouldn't have eaten that. It’s not anybody who's made me feel that way. I just always want to do good and I want to make sure things go well. And then I think I put that pressure on myself.” (Katrina, T2D)

Women pregnant for the first time may enter the pregnancy without adequate diabetes preconception counseling. The information could be new and overwhelming, compounding distress. There was a significant shift in Caitlyn’s diabetes management when she needed to familiarize herself with insulin administration and monitoring blood glucose. In addition to facing a learning curve to improve her competency, Caitlyn saw a medicalization of her pregnancy with higher insulin doses and taking time off work for more appointments. She felt unprepared for the shift she experienced in how to manage her diabetes.

“And it’s been a huge learning curve. Previous to this, I've never had to use insulin. And I didn’t really test my blood sugars, I was pretty well balanced with using just metformin. It's been a huge change and shift in the fact that things have really escalated in the amount of insulin, number of doctors’ appointments, missing work.” (Caitlyn, T2D)

#### Change to the presentation of diabetes

It could be difficult to adapt to the physical manifestation of diabetes changes that occur during pregnancy, as seen with blood glucose variability and insulin resistance. As insulin resistance increased, exogenous insulin administration increased to combat the concurrent blood glucose rise. Lisa (T1D) recalled taking “*double, may even triple the amount of insulin*” in her third trimester for stubborn blood glucose which left her with a “*defeated*” feeling and that the insulin resistance was not going to end. This sense of being defeated by diabetes was also shared by Saara (T1D), who grew frustrated with the variability of her daily blood glucose and when her usual insulin correction and hydration strategies to address a high glucose did not work. This would result in days when she would skip eating a snack, feel hungry and tired, and be*“running high all day and then just feeling lousy on top of being very pregnant.”*

Conversely, increasing levels of insulin needed to be carefully balanced against the risk of hypoglycemia. Hypoglycemia was a fear because of the frequency and severity of the lows that women were experiencing. These rapid blood glucose drops were hard physically and emotionally. For Barbara, there was a fear associated with overnight hypoglycemia since women did not trust that they would wake up in time to feel and treat the symptoms. Barbara shared her dread:

“I still wake up afraid that I won't wake up, that kind of thing. Cause I'm having hypoglycemia during my sleep.” (Barbara, T1D)

### Theme 3: Living with diabetes –* “There’s no way out”*

Many participants spoke to an aspect of their lived experience with diabetes that was complicated by negative or isolating interactions, stigmatizing experiences in which diabetes was misunderstood, and difficult internal feelings in relation to pregnancy. Ignoring diabetes while pregnant was not possible due to the nature of this chronic, always-demanding condition.

#### Unable to escape diabetes

As a chronic condition, diabetes could not be paused for a break, which is particularly true during pregnancy. Some participants felt the burden of the condition in how it takes a toll on their bodies, complicates their pregnancy, and leads to feeling like their whole pregnancy revolves around blood glucose management. Adelaide spoke of how she found it impossible to shed the high-risk label in her pregnancy:

“There's no way out of it. There's no way even though I really tried my hardest to have good numbers, I did everything I could for the baby to be healthy and happy, you’re no longer not high risk anymore. So you could do everything you possibly could do and you could still be ‘risky’. I think that word risk is just scary.” (Adelaide, T2D)

Adelaide was working hard to do the best she can, but because her diabetes never goes away, the high-risk label does not go away. She was frustrated that all her efforts still make her feel like she falls short, and the high-risk terminology will always make her feel like her baby’s health is in jeopardy. Diabetes preoccupied the mind and its omnipresence changed the perception of the pregnancy experience. Rachel found diabetes was always on her mind during pregnancy:

“Honestly, you have to make changes every single day and you're having so many lows, so I feel like diabetes was kind of like the forefront of everything in my pregnancy and worrying about it because I was diabetic.” (Rachel, T1D)

Ruth echoed the sentiment that diabetes was an ever-present burden, defining her pregnancy:

“It felt like my whole pregnancy revolved around diabetes because of everything else I had to do for the diabetes during my pregnancy. When I look back at my pregnancy that's what I think of.” (Ruth, T2D)

Here we saw that Ruth’s recollection of her pregnancy was done through a lens of diabetes. This shift in perspective was because of the extent that diabetes took over her life while she was pregnant. Barbara (T1D) displayed a dichotomy of emotions, signaling she felt failure while minimizing the impact of diabetes had on her: “*Yeah, a little bit of a burden. Like that I couldn't hold my own weight.”*

In addition to this mental burden, living with pre-exisiting diabetes in pregnancy had a physical burden on the body, including fatigue and mood swings, as shared by Lisa:

“Just that diabetes in general, but especially during pregnancy, can take quite a toll on your body. So it is exhausting. And there are mood swings that come with high and low blood sugar swings.” (Lisa, T1D)

Women saw their diabetes confining them and depleting their mental and physical energy that they wished they could direct into other aspects of their pregnant lives.

#### Challenging interactions with others

Negative interactions with others could include family members, healthcare providers, colleagues, or strangers. These interactions were characterized by hurtful comments, judgments, or misperceptions about their diabetes. Insensitive comments spoken to the participants often had lasting effects, as Saara described:

**“**I remember again having the discussions [about effects of hypoglycemia] with Dr. C who, I mean I know he's a very intelligent man, he doesn’t get an A plus in bedside manner. Because I remember him saying ‘large and floppy babies'. I remember like it's ingrained in my head, that was his answer. And it created more anxiety within me, I don't know what that means.” (Saara, T1D)

In this exchange, Saara was given information she did not understand in insensitive language. She did not feel that her care provider was able to communicate with her in a productive way or in an emotionally supportive way. As a result, the worries she had about the effects of hypoglycemia expanded to include anxiety about her baby being abnormal.

Adelaide also experienced how comments made by others were not constructive and instead caused new worries and anxiety during her pregnancy. She received judgmental comments from her in-laws about diabetes causing babies to be very large, which preyed on an existing fear she had. Feeling this type of judgement, lack of understanding, and liberties to speak on aspect of diabetes they did not understand was a common experience for the participants. Misperceptions about how one should control their blood glucose or the complications of diabetes could be expressed by close family members, adding another layer of frustration for women with diabetes. In some cases, even close family members may not truly understand what the participants went through with their diabetes. Barbara shares that her diabetes was not a straight line she could always follow:

“And trying to explain that [every day is different with diabetes] to people sometimes that are non-diabetics, they don't necessarily understand that. And even my mother, who's lived with me for years, she doesn't understand that there's not a day that's the same.” (Barbara, T1D)

For Delilah, the experience of misunderstanding diabetes was verbalized during a medical interaction, causing her distress and highlighted the ignorance of others:

“I went for bloodwork during pregnancy and I was six months pregnant. And the lady at the clinic told me, like ‘Aren’t you worried that your baby is going to have diabetes because you have diabetes?'. There were people that are just very ignorant about things. And I remember leaving there crying, because I'm six months pregnant, there's really nothing I could do about it. And also it's not helpful.” (Delilah, T1D)

Stigmatizing experiences could happen before or during pregnancy and these experiences were not limited to certain individuals. Women shared passages describing a range of people, from family to providers, to co-workers who contribute to diabetes stigma, which occurred at a time when women feel vulnerable and worried in their pregnancies. Barbara shared negative social judgment she received about blood glucose monitoring in the presence of coworkers:

“Some people at work weren’t supportive, some of them didn't understand because they didn't understand the condition. For example, some people got offended if I took my glucometer out and tried to test instead of in the bathroom.”(Barbara, T1D)

Similarly, some participants felt a personal stigma attached to their high-risk pregnancy status. Adelaide shared her view of this label and how it shaped a negative outlook of the pregnancy:

“Just being deemed high-risk is in and of itself stressful. I look at it as a higher risk meaning it not being successful, right? I think that is worrisome. I would much rather be considered low risk with a better success rate or no deformities. I worried about losing the baby that first six weeks, where was it as far as development goes? Was I at the point of going to lose it? Was it going to be a viable pregnancy? And knowing it was high-risk, that meant I need more monitoring which is stressful, which is time consuming.” (Adelaide, T2D)

Lastly, some participants received negative messaging about pre-existing diabetes in pregnancy from others which emphasized worst-case scenarios about the baby’s health. This often came from trusted professionals or close family members and these comments could be unexpected and hurtful. Rachel (T1D) disclosed that her mother “*didn't want me to have a baby shower until after the baby was born because I'm diabetic. And basically, the baby might not make it.”* As a result, her pregnancy was not celebrated in the same way as someone else because her mother felt it would be premature to hold a shower. Justine felt shame and blame for her unplanned pregnancy from her family doctor:

“And then when I did go to her and we tested my sugars, finally, she told me basically that it's going to be a brutal pregnancy, that there's a good chance she might be a stillborn. Not sure if she’ll even survive, or going to have so many health issues. And she basically just blamed me for all of that. I was really scared from that. And it made it really difficult for me to have motivation to do better for the pregnancy, because she almost made it sound like there was no hope for me to have a successful pregnancy.” (Justine, T2D)

Delilah recalled her experience in the pediatrics diabetes clinic and learning about the risks of pregnancy, which led her to have life-long anxiety about becoming pregnant:

“Also, when I was diagnosed at five years old, and when I was in the pediatrics clinic, I was told at that point that women died from trying to have babies being diabetic and you might not ever be able to have a baby. It was kind of one of those things where I was so worried about it, because my whole life I worried if I was even going to be able to have a baby, that I didn't want anything to happen.” (Delilah, T1D)

For Delilah, this message was the worse outcome for a woman with diabetes who wanted to have a family: it could not happen, and her life was at risk if she tried. She shaerd how the the long-term impacts of hearing this influenced how she approached romantic partnerships.

#### Diabetes restricting or changing the experience of pregnancy

The perception that a pregnancy with diabetes was different from a typical, low-risk pregnancy was articulated because it was becoming more medicalized with increased visits, assessments, and testing. Alex describes how this contrasted against her first pregnancy:

“I was very nervous about being diabetic and pregnant. With my first pregnancy I didn't have to have a lot of ultrasounds…I was really grateful that there was so much, attention to the to the baby and how her health was and having all those ultrasounds. I knew I was doing the right thing but it still felt like it was a little bit against how I pleasantly envisioned it going [laughs]. A lot more medical intervention than the first time around, so it was just getting used to that.” (Alex, T2D)

Adelaide struggled with accepting a caesarean section delivery rather than her dream birth plan of a natural delivery because of being high-risk:

“I think the feedback from them scared me a lot. Knowing just from my previous experience and how I handle it, and especially this time around because I had been given the hope of having a [vaginal birth] and I clung to it but I was scared of having too much invested in that desire to have a natural childbirth. So, the C-section scared me and I felt like diabetes was responsible for the C-section. And that I was going to lose control over that desire to have that experience in my life. Because for whatever reason I had put a lot into having a baby naturally and experiencing that. And I know no matter when people say like a birth is a birth, however the baby comes out is the safest way for the baby. But, the thing that I really wanted was an advocate and a peaceful, calmer birth.” (Adelaide, T2D)

This passage revealed the desire women have for a “peaceful, calmer birth”. They would fantasize about an uncomplicated delivery and hope they could avoid medicalization of the births of their baby. Diabetes could also result in restrictions on behaviours thought to be average and healthy in pregnancy. Delilah experienced a major shift to her routine when she was instructed not to exercise due to frequent hypoglycemia:

“Before I got pregnant, I was working out about five times on average. I had so many lows the doctors told me not to work out anymore. So there were a lot of things that I did before that normally are good for your health of your baby and the health of you during pregnancy that you just can't do when you have this kind of added issue of diabetes involved in it.” (Delilah, T1D)

For some women, not knowing others with pre-existing diabetes in pregnancy or having limited peers with diabetes in whom to confide or ask questions leads to feelings of isolation. Some participants felt alone in their diabetes and pregnancy experience. They desired to relate to others and know that others have been in the same situation and experienced good outcomes. Kelsey found it difficult to discuss the challenges she had with her diabetes to her partner:

“Is your husband supposed to be your main support system? But if he's the kind of a person who doesn't want to hear anything bad or anything’s bad happening and stuff, who shuts down…you feel alone in a way, right?” (Kelsey, T2D)

This showed the need to confide in people other than a partner. Kelsey indicated a preference for connection with her female relatives who could understand the broad experience of pregnancy, but her limited support system in Canada contributes to her feelings of isolation. She commented that a virtual connection would not be the same as in-person support. Giselle interacted with a virtual community of women with diabetes so she had a safe space to ask questions because of the limited real-life connection she had with other women with diabetes. She acutely felt the difference in her pregnancy with diabetes compared to the women she knew who had low-risk pregnancies:

“I have one friend that's type 1 diabetic. And she's not a really close friend. I don't know anybody else in my close group or family that's a diabetic, so it's kind of like you're going through this by yourself. You can talk to somebody who's not a diabetic and who's pregnant, but you're not going through the same thing. It's a very different pregnancy. So that was that was the best outlet that I had.” (Giselle, T1D)

The pre-existing diabetes in pregnancy clinic was an obvious but underutilized source of connection to combat this feeling of loneliness. Despite attending the clinic with other women who had diabetes, Adelaide felt alone due to the lack of interactions between the patients:

“There's this entire clinic meant to be helpful and it's a good thing, but it's overwhelming when you're the one sitting there, staring at the walls at all these posters in a waiting room or in the doctor's office with no one to talk to.” (Adelaide, T2D)

#### Guilt about having diabetes while pregnant

Women often spoke about feeling guilt around their diabetes, which included blaming themselves for their diagnosis, taking responsibility for the potential effects of diabetes on their baby’s health, and feeling guilt-ridden that they were not doing enough to protect the baby’s health with their blood glucose management. This was sometimes thought of as a failure on the part of the participant. Some women expressed guilt over past pregnancy outcomes they felt were due to their diabetes.

Adelaide and Alex spoke about feeling like it’s their fault that they have a type 2 diabetes diagnosis. Adelaide felt badly she is now subjecting her baby to her diabetes:

“The entire thing is stressful. [It comes with] a lot of guilt, a lot of regret. I personally blame myself for having diabetes. But that comes along with a lot of guilt during a pregnancy of, you know, I did this to myself. I now have to put this poor baby through a chance a of something.” (Adelaide, T2D)

Alex questioned if her desire for a family was fair when she could be putting the baby at risk:

“I mean, I'm type 2 diabetic because of my family history but just also because of my lifestyle. I want to have children, but then I feel guilty that's my want but is it fair to be pregnant and possibly putting that child, in danger for that length of time while I’m pregnant?” (Alex, T2D)

Both of these women felt that because of diabetes, they may not be entitled to the same life experiences such as pregnancy as someone else. They viewed their diabetes as something they have done wrong, and so their guilt is directed at themselves.

There was guilt about both the potential or actual pregnancy outcomes because of pre-existing diabetes in pregnancy. Delilah felt burdened by thoughts about the risks of hypoglycemia she was experiencing on her baby’s development:

“There’s not as much research on what low blood sugars do to your body as opposed to high blood sugars. But we do know that low blood sugars can kind of deplete brain cells. So, when you're growing a baby who's developing its brain and spinal cord and then you're having lows, you feel like you're not able to give the baby what it needs necessarily to develop properly. So you worry a lot about that kind of thing and you're doing your best, but at the same time you feel out of control and like you're not able to be the best mom for your baby already.” (Delilah, T1D)

Alex shared her feelings of guilt about her miscarriage and her embarrassment that others may blame her diabetes on the loss:

“I found it scary and many people didn't also know that I was diabetic? It isn’t something that I feel like I have to share, also just a little bit embarrassing to the people that did know that maybe the first loss was sort of my fault? And I had that fear that that was going to happen again.” (Alex, T2D)

These women carried a large burden of guilt and shame for their diabetes that left them feeling inadequate and at times deserving of the stressful experiences they have.

### Theme 4: Cycle of diabetes distress

**Fig. 1:**
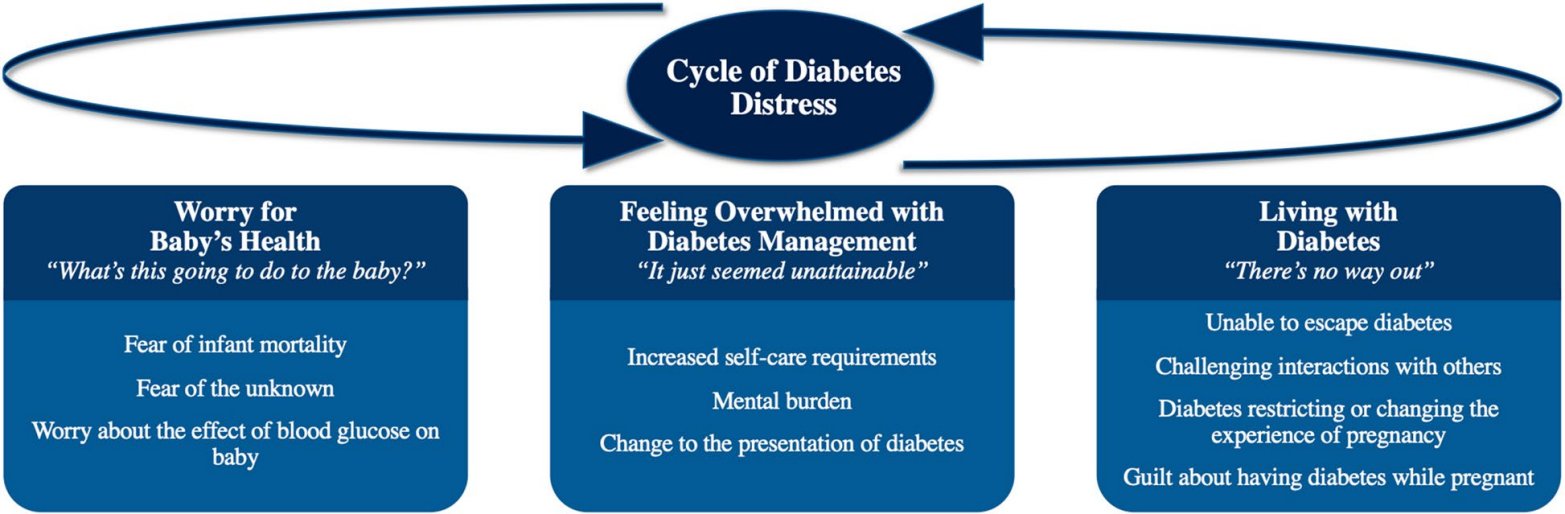
Themes and cycle of diabetes distress

The three themes of DD described above interact together across the timeline of pregnancy (Fig. 1). As pregnancy progresses, there may be a temporality seen in the themes of distress that emerge and the way they feed into each other, leading to the DD cycle. For example, in the first trimester, hypoglycemia was commonly experienced which could be difficult to manage and lead to fears about both the mom and baby's safety. To mitigate these harms, women could increase how often they were testing their blood glucose, which brought further burden and the perception their pregnancy was being overshadowed by diabetes. This led to more negative feelings such as stress and frustration with how much effort their diabetes required.

When Saara (T1D) was experiencing this in the first trimester, she remembered sharing her fears with the healthcare team, saying: *“Look, I can't feel them [low blood sugars]. I'm not going to wake up from my sleep and something's going to happen.”* To reduce the risk of a severe low occurring and causing harm to her and the baby, Saara began taking more food at night without insulin to ensure she wouldn’t drop. She recalled her endocrinologist “*wasn’t extremely pleased*” with her blood glucose numbers, but could only provide her with the suggestion to test frequently, which was not helping the frequency of her lows. 

In the second trimester, women started to experience insulin resistance which leads to high, unpredictable blood glucose. They worried about the effects of hyperglycemia on their baby’s health and size and work to manage the high glucose through adjusting their insulin, improving carbohydrate counting, and testing. Caitlyn found her blood glucose had a lot of variability, which made them hard to control. This led to anxiety about how this would affect her baby:

“Well, not necessarily [worry about] myself, but more like a complication that will happen with the baby. And the baby being on the larger side, and the risk of stillbirth late in pregnancy with diabetes. Those things are more of my concern than anything else.” (Caitlyn, T2D)

This regimen led to feeling like diabetes was overshadowing the pregnancy, which did not feel like a “*typical*” pregnancy. For Caitlyn, she felt “*scolded*” by a healthcare team member for being ignorant of the complications of pre-existing diabetes in pregnancy. Ruth (T2D) felt like she was “*failing at the interventions”* because the healthcare team had to keep increasing her insulin to manage her high blood glucose. Other participants reported how the variability of their blood glucose led to feeling mentally and physically exhausted.

An example of the distress cycle in the third trimester was being unable to reach desired blood glucose targets after making dietary adjustments, counting carbohydrates more accurately, and doing more blood glucose testing. There were feelings of anxiety from putting in this effort but still frequently seeing high glucose results, as demonstrated by Giselle:

“Finding out my insulin ratios, what foods would go well with maintaining good blood sugar and being always being cognizant that my blood sugars could affect my baby. So that was probably the most stressful thing to go through was…if I ever saw high blood sugar I would think I'm hurting my baby. It was just it was a very emotional toll.” (Giselle, T1D)

Meetings with the diabetes care team more frequently to address the glycemic results could feel judgmental and lead to feeling guilt and pressure to improve the blood glucose in a short period. Giselle (T1D) found that sometimes the interactions with care providers would leave her feeling guilty about her blood glucose, and suggests providers could give *“just a little bit more feedback on what you can do to help versus making them feel not so great about not hitting those targets all day, every day.”*

Conversely, cycles of DD were seen to intermingle regardless of the trimester-specific timing. Many of the participants emphasized the level of work that was needed to manage their diabetes for their baby's health, and the sheer level of effort required from the person living with diabetes could be considered distressing on its own. Considered in the broader context of transition, this left some participants feeling vulnerable and not in control of their pregnancy which could lead to feelings of distress being exacerbated during negative interactions with others. There could be intensification of DD for a woman who had experienced loss in another pregnancy as she felt additional stress and worries that a poor outcome would be repeated despite her best efforts at self-management.

This is seen with Barbara, who had hypoglycemia unawareness at the beginning of her pregnancy. Because she was no longer feeling the signs and symptoms of low blood glucose, she began using continuous glucose monitoring as a safety measure. She became very anxious about the effect of any abnormal blood glucose she would see on her monitor because she was terrified of anything happening to her baby after experiencing a previous stillbirth.

“Because every single time I would have one [low blood sugar], unfortunately, I would have a little post-traumatic and thinking that something's happening to [daughter].” (Barbara, T1D)

#### *Description of figures: the transformative and emotional journey of pregnanc*y

**Fig. 2:**
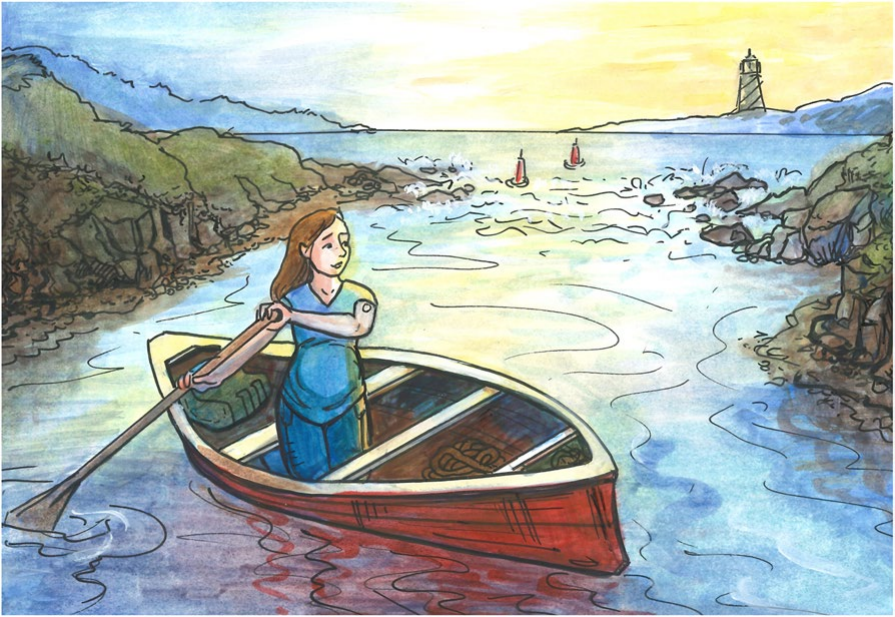
Time-Limited Pregnancy Journey

**Fig. 3:**
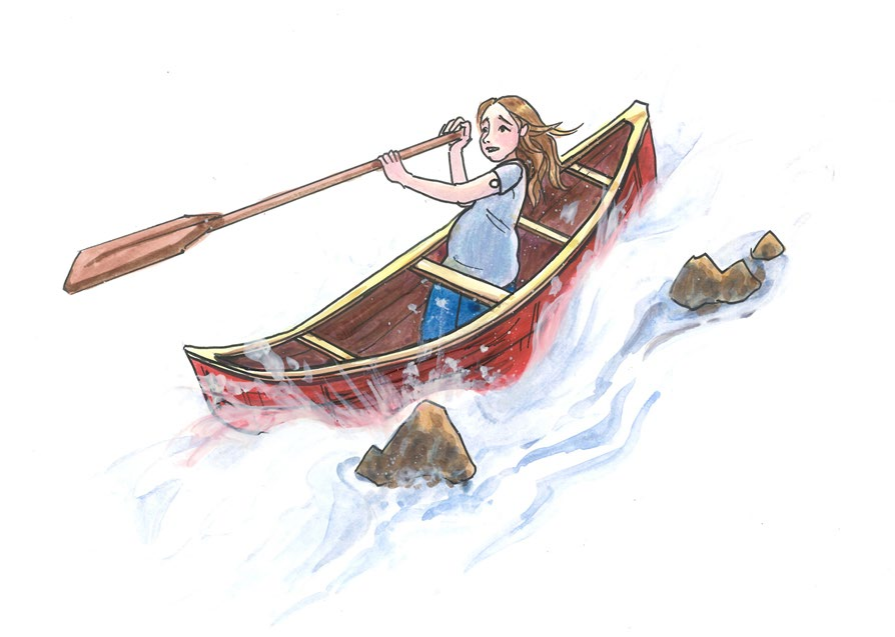
*Diabetes Distress*

To visualize and integrate the thematic elements of this emotional and physical transformative journey through DD and pregnancy, the illustrations of Figs. 2 and 3 have been created.

The scene in Fig. 2 illustrates the Time-Limited Pregnancy Journey by depicting a pregnant woman with diabetes flowing down a river in a rowboat. As she plans to move from left to right in the scene, her journey down the river goes from smooth, calm waters into rough, turbulent river water. The change from calm to stormy symbolizes the transition at the beginning of pregnancy when the physical and emotional aspects of diabetes drastically shift and the woman takes on a new form of diabetes for the duration of her pregnancy.

In Fig. 3, the woman is navigating her boat through rough river water, the worry evident on her face. There are rocks under the surface that cause the current to speed up, change direction, and create waves. These rocks under the surface represent the often-unseen aspects of pre-existing diabetes in pregnancy that cause distress for women, and are summarized by the four overarching themes of the finding *Diabetes Distress*.

There may be times throughout this journey that women risk going overboard by the waves and current, which is a time they are experiencing DD. Going overboard can happen anytime throughout pregnancy and sometimes a woman may risk going overboard more than once throughout her journey down the river. Eddies, or whirlpools, are formed as the water runs into the obstruction of the rocks. These represent the “Cycle of Diabetes Distress”. In a cycle of diabetes distress, one element of DD can build and lead to another, interacting together and creating an on-going cycle that can keep them experiencing DD.

Throughout pregnancy, women are riding these waves of emotion towards their ultimate guiding light and beacon, the lighthouse seen in Fig. 2. The lighthouse represents the common goal all the participants have: delivering a healthy baby. Each woman is willing to brave these rough waters to get to this destination. As they move forward in time, through the trimesters to delivery, their ultimate goal is a healthy baby and is depicted as a bright lighthouse in the distance. The lighthouse is their focal point, and guides them through the turbulent waves.

The figures represent the linear movement through the trimesters of pregnancy. Due to the nature of pregnancy, time cannot be paused or the course reversed; each woman is constantly moving forward. Because of the finite pregnancy journey, women are bound by the element of time. Each woman is willing to brave these rough waters for this fixed amount of time to get to their final destination.

## Discussion

In summary, the three themes of DD identified Worry for Baby’s Health – *“What’s this going to do to the baby?”*, Feeling Overwhelmed with Diabetes Management – *“It just seemed unattainable”,* and Living with Diabetes – *“There’s no way out”* have the potential to interact with one another, leading to cycles of DD. In a cycle of DD, the feelings of distress build off one another and risk becoming a continuous feedback loop of negative emotions. Qualitative themes from interviews with 18 women with pre-existing diabetes revealed important insights into DD, supporting that women in pregnancy experienced DD and helps characterize the sources and experiences of DD. Pre-existing diabetes in pregnancy was seen as a transformative, emotional journey for women with T1D and T2D. At the center of this experience was the concept that pregnancy necessitates living with a unique, intensive version of diabetes. Each woman was confronted by an unavoidable and potentially unfamiliar form of diabetes to which they had to immediately adapt to have a healthy pregnancy. A physical and emotional transition took place as women rose to the challenge of stepping into and navigating a new world of diabetes, one which exceeded their usual management. This physical and emotional transition was defined by several characteristics, specifically the medicalization of pregnancy, increasing demands to self-manage diabetes, and physiological changes to the body. These elements subsequently planted the seeds for DD to arise as women navigated the transition.

There have been several qualitative studies that have explored the emotional experience of pre-existing diabetes in pregnancy, and they echo the accounts of DD seen in this study. The themes uncovered from these studies signal the presence of emotional and psychological distress that are frequently linked to the specific burden of diabetes. This includes the intensification of self-management required of diabetes within the context of pregnancy and the shame and fear women feel about the effect their blood glucose management will have on the health of their babies [19, 20, 38–42]. Despite the majority of the above studies not referring to these experiences using the now well-established term of DD [19, 20, 38–41], it could be argued that the findings did identify the experience of DD in pregnancy. For example, Singh et al. [40] identified subthemes of specific diabetes self-care tasks (monitoring blood glucose, following a diet and carbohydrate counting) that felt so restrictive the women felt they were not able to enjoy their pregnancies.

There are also profound similarities in subject matter and even terminology across these studies compared to our participant accounts. For example, the following quote from Edwards et al. [42] described a women’s worry about high blood glucose during pregnancy that mimics the stories of anxiety and guilt that was explored in Theme 3: Living with Diabetes – *“There’s no way out”* when blood glucose management was frequently seen at the forefront of participant’s minds during pregnancy*:*

*“I have been so stressed and racked with guilt whenever I have higher levels, and it has only been 3 weeks – I will be a nervous wreck by the end of the pregnancy!”* [42]

Interestingly, the study by Edwards et al. identified DD in their thematic analysis of the pregnancy journey of women with T1D as an important component of mental health and emotional well-being [42]. The authors noted that DD, which was coded from written interactions, was expressed differently in pregnancy compared to when it was discussed during the contemplation of motherhood, centering around increased anxiety related to the baby. While there was limited detail provided about this theme overall, it supports our finding that DD in pregnancy is intricately linked to the baby’s outcomes.

The focus on the physical body and how that can lead to negative emotions were seen across the first three themes in this study. Pregnancy may be the first time that a woman has to acknowledge the complications of diabetes, which in pregnancy can worsen or manifest in a short period and have tangible consequences for the mother and baby. While it is established there are better outcomes with lower blood glucose levels [1], complication frequency remains elevated in this population despite pharmacological and technological advances in blood glucose management. For example, rates for congenital anomalies (relative risk 1.86 [95% CI 1.49–2.33] and perinatal mortality (relative risk 2.33 [95% CI 1.59–3.43]) that continue to be significantly higher compared to the general obstetrical population [10]. Our observation of how important it was to participants to avoid medical and diabetes complications was also seen in the 2013 literature review by Rasmussen et al. [38], which reported that women were driven to normalize blood glucose levels for both baby’s health and their own risk of complications.

Women also feel a lack of control of their body and their diabetes, noting how challenging it is to stay on track with their evolving diabetes. Their struggle for glycemic management leads to both physical and emotional fatigue, as women reported feeling exhausted by their efforts. They also felt frustrated that their body wasn’t cooperating at the time they need it to. Multiple qualitative studies detailed similar themes: an “unwell” body and “not knowing my own body” [19], losing control [38], physiological reactions being unexplainable and uncontrolled [20], and feeling vulnerable [43]. Risk to self and risk to baby (specifically if their child would inherit their condition) was a salient theme in the study by Singh et al. [40] and the findings of others mirrored what was described in our study.

Appropriate strategies are needed to address and minimize DD in pregnancy. The participants in this study found ways to minimize the DD they were experiencing during pregnancy, and this is discussed in the companion paper [32]. A qualitative meta-synthesis on the experience of psychological distress during pregnancy uncovered a specific transition towards motherhood that starts in pregnancy and was defined by grief and loss due to having an imperfect pregnancy [44]. In this study, participants voiced sadness and disappointment as the medicalization of their pregnancy changed their vision and expectations of this time in their lives. There was evidence that our study participants felt a similar vulnerability and dissatisfaction to be categorized as high-risk and require intensive medical care. Mindfulness-based interventions to enhance self-awareness, acceptance, and knowledge have been suggested to help managing feelings of lack of control and impending transition to being a mother [44]. There is also growing evidence on the benefits of mindfulness-based interventions to mitigate DD [45–47] however more research is needed to assess the effectiveness of interventions for DD in pregnancy.

Self-management for the different forms of diabetes is recognized to consist of not only physical and cognitive tasks [1] but also socio-emotional tasks, and ultimately these efforts are work [48]. All of these elements of self-management are influenced by a multitude of factors: economic, socio-cultural, workplace and community, social capital and psychological factors [48]. Individuals with diabetes have noted the “constant pressure” to meet blood glucose targets and they identified the need for emotional support from healthcare providers without the use of “scare tactics” around blood glucose control [40]. Creating an “obsessive” culture around glycemic targets and SMBG results in exaggerated feelings of guilt and frustration when blood glucose inevitably spikes up or down [40]. It is interesting to observe the similarity of language women in this study describe the same pressurizing experiences (under Theme 2: Feeling Overwhelmed with Diabetes Management—“*It just seemed unattainable*”). Therefore, providers need to consider using a more person-centred approach when delivering self-management education. With a need for medical information to mitigate feelings of risk to themselves while pregnant, there is a fine balancing act between consuming so much information it leads to feeling overwhelmed and anxious, versus feeling uninformed and unsupported by a lack of information [31]. The literature supports our finding that education must be comprehensive but thoughtful and sensitive to women’s needs in both diabetes and pregnancy with clear communication and points of contact within multidisciplinary teams.

There is a need to create and validate pregnancy-specific DD screening tools. Once DD is properly identified, interventions for DD could be incorporated into the clinical setting. A pragmatic trial randomized nearly 400 adults with T2D and positive DD and compared a web-based behavioural self-management program with or without DD-specific problem solving therapy to a general, computer-based diabetes support program [21]. Significant improvements in DD scores were found across all three arms (*p* < 0.001). The authors concluded that as DD was responsive to all interventions, DD may be reduced with healthcare provider support and attention with or without structured programs [21].

To address DD within the clinical care space, one review recommends that within a focused conversation with their healthcare providers, individuals with DD can be encouraged to reflect and label their feelings, beliefs and expectations using techniques such as active exploration, summarizing, normalizing, and double reflection [49]. This is followed by providing new information that helps identify and correct existing assumptions and expectations, leading to a new perspective on diabetes. These strategies could also be particularly useful in pregnancy when women feel emotional changes and immense pressure related to their glycemic management. The next steps in this process are to develop a focused plan that addresses small, feasible changes with frequent follow-up [49]. Seeing DD interventions as a process means building in the time for this type of follow-up, which occur frequently during pregnancy care but typically focus on physical health and blood glucose management rather than emotional health.

Diabetes health coaching models utilize brief but frequent follow up but there has been limited research into how this approach can be used to reduce DD as the primary outcome. A nurse-coaching pilot intervention that used educational, behavioral and affective strategies resulted in lower DD scores from baseline to 6 months in a sample of women with T2D (*F* = 7.5, *p* < 0.01) [50]. A randomized controlled trial of health coaching that is underway will utilize a behavioral and psychological management intervention that includes diabetes self-management education and support and a DD-specific education program delivered over the telephone for non-pregnant adults with T2D who screen positive for DD [51].

The coronavirus disease 2019 (COVID-19) pandemic has enhanced feelings of distress and anxiety in perinatal populations, particularly in the context of the loss of control. Feelings of loss of control and fears about health and safety were critical themes voiced by general perinatal populations studied in the context of the COVID-19 pandemic, during which feelings of distress and anxiety were found to be enhanced [52]. The COVID-19 pandemic highlights the importance to ensure adequate psychological resourcing is available to women to help manage DD which is even further elevated in the context of future pandemic lockdowns and restrictions. Including mental health professionals in the existing multidisciplinary team approach [1] in addition to utilizing diabetes educators for routine DD screening could be a way to assess and support psychological well-being during pregnancy [40].

Preconception planning is an optimal time to intervene proactively and will set up with women with knowledge and expectations of future pregnancies. Despite the importance and recommendations for preconception and pre-pregnancy planning in women with pre-existing diabetes [1, 2, 53–55], preconception care is frequently fragmented in its approach. Many pregnancies remain unplanned [55, 56] and women with T2D have been shown to receive less preconception care than women with T1D in Canadian studies [57, 58]. There is evidence of important differences between the pre-pregnancy care provided to women living with T1D and those living with T2D, with researchers highlighting a deficit of systems-level processes and healthcare provider attitude and engagement to integrate pre-pregnancy care into the usual diabetes care of women with T2D [59, 60].

Women with T1D report a strong preference for information related to pregnancy to mitigate their limited knowledge and fears related to pregnancy, including specifics on risks and the importance of glycemic management on outcomes [41]. Pregnancy planning is a time described as “emotionally complex” due to the worries and fears women already have for themselves, their future baby, and their ability to self-manage [41]. Preconception care generally focuses on improving glycemic management and folic acid dosing before conception to lower the likelihood of damage during organogenesis but there is also an opportunity to discuss the emotional challenges of pregnancy.

### Strengths and limitations

A limitation of this study is the small sample size of 18 women that were recruited. While saturation signals in the data showed adequacy and comprehensiveness of the information to answer the research questions, conducting further studies to confirm or elaborate on these findings would be useful. There were only four participants included who were in their first pregnancy, so their experiences may have differed from the women who had previous experience of managing diabetes during pregnancy and were able to develop either self-care strategies or resilience. The majority of women were postpartum, and therefore not currently experiencing pregnancy while being interviewed. While it could be a concern that more time elapsing since pregnancy results in a decrease in the recall of experiences, the richness of the study data demonstrates otherwise. All postpartum participants shared detailed, emotional accounts of their experiences of pregnancy. They were able to reflect meaningfully on the entirety of their pregnancy experiences and it did not appear that any recall had been compromised with distance from the experience.

A strength of this study was its aim to purposely explore DD, a known psychosocial construct, in pregnancy in women with both T1D and T2D. Interview probes specifically asked about experiences of DD with participants, exploring in depth how they perceived DD and broad aspects of self-management. To our knowledge, this is the first study to have this specific aim. Ten out of the 18 participants were women with T2D. Women with T2D have largely been left out of research about the qualitative experiences of pregnancy, which has focused on the voices of women with T1D. Their unique perspectives on the stressors of pregnancy are critical to capture as they are leading the rise in pregnancies complicated by diabetes [7–9].

## Conclusions

The findings from this study suggest that women with pre-existing diabetes commonly experience DD throughout their pregnancies. A physical and emotional transition takes place as women rose to the challenge of stepping into and navigating a new world of diabetes that exceeded their usual management due to: the medicalization of pregnancy; the increasing demands to self-manage diabetes; and the physiological changes to the body. Sources of DD include specific worry for the baby’s health, feeling overwhelmed by diabetes management, and the lived experience of diabetes that is a constant presence in their reality. The themes of *Diabetes Distress* describe the unique sources and presentations of DD while pregnant, which were found to be experienced in a cyclical nature throughout pregnancy.

Future work should continue to examine DD in different pregnancy populations from both a qualitative and quantitative lens, including examining how DD is measured in pregnancy, the impacts of distress on neonatal and delivery outcomes, and clinical support and management strategies that can mitigate DD. Importantly, the experience of pregnancy for the group of women was not all negative because they found ways of managing DD. As this current research produced a second qualitative finding, the companion paper will describe in detail the sources of resiliency accessed by these women with pre-existing diabetes to cope with DD.

## Data Availability

The dataset analysed during the current study are not publicly available due to subject confidentiality, but are available from the corresponding author on reasonable request.

## Abbreviations

DD: Diabetes distress
T1D: Type 1 diabetes
T2D: Type 2 diabetes
SMBG: Self-monitoring blood glucose
PAID: Problem Area in Diabetes Scale
COVID-19: Coronavirus disease 2019
